# Final results from the phase Ia/Ib study of the novel bromodomain and extra-terminal domain inhibitor, BI 894999, in patients with advanced solid tumors or diffuse large B-cell lymphoma

**DOI:** 10.1016/j.esmoop.2025.104499

**Published:** 2025-04-08

**Authors:** U.M. Lauer, A. Awada, S. Postel-Vinay, G.I. Shapiro, C. Thieblemont, S.A. Piha-Paul, P.K. Paik, D.R. Shepard, L.I. Docampo, R. Galot, S. Rottey, B. Sadrolhefazi, K. Marzin, H. Musa, P. Schöffski

**Affiliations:** 1 Department of Internal Medicine VIII, Medical Oncology & Pneumology, University Hospital Tübingen, Tübingen, Germany; 2 German Cancer Research Center (DKFZ), Member of the German Cancer Consortium (DKTK), Tübingen, Germany; 3 Oncology Medicine Department, Jules Bordet Institute, Brussels, Belgium; 4 Drug Development Department (DITEP), Gustave Roussy, Villejuif, France; 5 INSERM Unit U981, Gustave Roussy, Villejuif, France; 6 University College of London Cancer Institute, London, UK; 7 Department of Medical Oncology, Dana-Farber Cancer Institute, Boston, USA; 8 Université Paris Cité & Assistance Publique-Hôpitaux de Paris (AP-HP), Hôpital Saint-Louis, Hémato-oncologie, Paris, France; 9 Department of Investigational Cancer Therapeutics, University of Texas, MD Anderson Cancer Center, Houston, USA; 10 Thoracic Oncology Service, Department of Medicine, Memorial Sloan Kettering Cancer Center, New York, USA; 11 Department of Hematology & Medical Oncology, Cleveland Clinic, Cleveland, USA; 12 Lung and Head and Neck Cancer Unit, Medical Oncology Department, University Hospital 12 de Octubre, Madrid, Spain; 13 Department of Medical Oncology, Institut Roi Albert II, Cliniques Universitaires Saint-Luc, Brussels, Belgium; 14 Institute for Experimental and Clinical Research (IREC, pôle MIRO), Université Catholique de Louvain (UCLouvain), Brussels, Belgium; 15 Drug Research Unit Ghent, Ghent University Hospital, Ghent, Belgium; 16 Boehringer Ingelheim Pharmaceuticals Inc, Ridgefield, USA; 17 Boehringer Ingelheim Pharma GmbH & Co. KG, Biberach an der Riß, Germany; 18 Boehringer Ingelheim International GmbH, Ingelheim am Rhein, Germany; 19 Department of General Medical Oncology, University Hospitals Leuven, Leuven Cancer Institute, Leuven, Belgium

**Keywords:** bromodomain and extraterminal domain inhibitor, diffuse large B-cell lymphoma, solid tumor, dose escalation, dose-limiting toxicities

## Abstract

**Background:**

Bromodomain and extraterminal domain (BET) inhibitors have demonstrated efficacy in solid and hematological malignancies. BI 894999, a novel, orally administered BET inhibitor, has demonstrated preclinical efficacy.

**Methods:**

This was an open-label, dose-finding study evaluating BI 894999 for diffuse large B-cell lymphoma (DLBCL; phase Ia extension) and solid tumors [colorectal cancer (CRC), nuclear protein in testis (NUT) carcinoma, metastatic castration-resistant prostate cancer (mCRPC) and small-cell lung cancer (SCLC); phase Ib cohort]. The primary endpoint was dose-limiting toxicities (DLTs) during the maximum tolerated dose (MTD) period (phase Ia) and treatment period (phase Ib).

**Results:**

Eighteen patients with DLBCL were enrolled in the phase Ia extension and 79 with solid tumors in phase Ib cohorts (SCLC, *n* = 12; CRC, *n* = 14; mCRPC, *n* = 11; NUT carcinoma, *n* = 42). Four patients had DLTs in phase Ia and 17 in phase Ib; the most frequent was grade 4 thrombocytopenia. The MTD for DLBCL was 1.5 mg (days 1-14/21). One patient (5.6%) with DLBCL achieved a partial response (PR) and three (16.7%) had stable disease. Of 42 patients with NUT carcinoma, 3 patients (7.1%) had responses (complete response, *n* = 1; confirmed PR, *n* = 1; unconfirmed PR, *n* = 1). Responses in other solid tumor types (*n* = 37) included one patient (2.7%) with mCRPC who had a confirmed PR.

**Conclusions:**

The safety profile of BI 894999 was consistent with those of other BET inhibitors. Due to minimal efficacy results, further evaluation of BI 894999 as monotherapy is not planned.

## Highlights

• BI 894999 is a novel BET inhibitor that has demonstrated preclinical efficacy.
• This open-label, dose-finding study evaluates BI 894999 for diffuse large B-cell lymphoma (*n* = 18) and solid tumors (*n* = 79).
• 4/17 assessable patients had DLTs in phase Ia and 17/79 in phase Ib; most frequent was grade 4 thrombocytopenia.
• Maximum tolerated dose was 1.5 mg for diffuse large B-cell lymphoma; safety profile was in line with other BET inhibitors.
• BI 894999 did not demonstrate sufficient efficacy to warrant further investigation.

## Background

The bromodomain and extraterminal domain (BET) protein family, including BRD2, BRD3, BRD4, and BRDT, comprises epigenetic readers that regulate gene transcription by binding to acetylated lysine residues on histones and master transcriptional factors.^1^, ^2^, ^3^, ^4^, ^5^ BET proteins may be involved in pathological conditions, including tumor development.^6^ BRD4 shows preferential localization to some superenhancers, which collectively facilitate transcription and strongly promote expression of oncogenes and master regulator genes.^6^, ^7^, ^8^, ^9^, ^10^, ^11^ Inhibition of BET proteins, especially BRD4, can exert a selective effect on the expression of specific genes in malignant cells, resulting in antitumor activity.^12^^,^^13^ Consequently, BET inhibition is a potential treatment strategy for several cancers.^10^^,^^14^

BI 894999 is a novel, orally administered, selective BET inhibitor, with demonstrated efficacy *in vitro* and *in vivo*.^13^^,^^15^^,^^16^ This small molecule inhibits protein–protein interactions between a histone H4-derived peptide with acetylated lysines and the bromodomains BRD4-BD1 (IC_50_ 5 ± 3 nM) and BRD4-BD2 (41 ± 30 nM).^13^ BI 894999 has demonstrated activity against colorectal cancer (CRC) cell lines^16^ and tumor regressions in nuclear protein in testis (NUT) carcinoma xenograft models.^13^

BI 894999 has been evaluated in a phase Ia/Ib, open-label, dose-escalation and dose-expansion study (NCT02516553). In the initial phase Ia dose-escalation study, three escalating dosing schedules were evaluated: schedule A was a dose-escalation regimen consisting of BI 894999 0.2 mg, 0.5 mg, 1.0 mg, 1.5 mg, 2.0 mg, and 5.0 mg given once per day on days 1-21 of a 3-week cycle; schedule B was an intermittent dosing regimen consisting of BI 894999 1.5 mg, 2.0 mg, and 2.5 mg given once per day on days 1-14 of a 3-week cycle; schedule C was a higher loading dose of BI 894999 (5.0 mg, 6.0 mg, or 7.0 mg) on day 1 followed by 6 days of a lower maintenance dose (2.5 mg, 3.0 mg, or 3.5 mg), repeated every 2 weeks in 4-week cycles.^17^ For solid tumors, the 1.5 mg dose in schedule A and 2.5 mg in schedule B were declared as maximum tolerated doses (MTDs), based on incidence and safety evaluations. The data monitoring committee declared the 7.0 mg loading dose and 3.5 mg maintenance dose (7.0 mg/3.5 mg) as the MTD for schedule C. However, based on dose-limiting toxicities (DLTs) reported during the MTD evaluation period, the MTD was adjusted to 6.0 mg/3.0 mg for schedule C in patients with solid tumors. Grade ≥3 DLTs occurred in 22 of 77 patients, with the most frequent being thrombocytopenia, increased troponin T, and decreased appetite. Disease control, defined as best overall response (BOR) of complete response (CR), partial response (PR; minimum duration of ≥14 days), or stable disease (SD; minimum duration of ≥39 days) by investigator’s assessment, was achieved in 26.0% of patients. The best response was PR, which was reported in 3.9% of patients.^17^

Phase I trials evaluating other BET inhibitors have reported promising results in patients with lymphoma, particularly diffuse large B-cell lymphoma (DLBCL). These trials reported responses in patients with DLBCL and other advanced hematological malignancies.^18^, ^19^, ^20^ Therefore, the phase Ia study was amended to evaluate BI 894999 in patients with DLBCL, where dose escalations were conducted on schedules B and C.

The phase Ib expansion evaluated BI 894999 in patients with advanced solid tumors, including CRC, NUT carcinoma, metastatic castration-resistant prostate cancer (mCRPC), and small-cell lung cancer (SCLC), using the MTD of schedule B and schedule C. BET inhibitors were developed as a potential treatment for NUT carcinoma, which is driven by BRD-NUT fusion oncoproteins, most commonly BRD4-NUT. Myelocytomatosis viral oncogene homolog (*MYC*) is a key downstream target of BRD-4 NUT in driving growth and blocking differentiation.^21^ The first-in-human study of molibresib, an orally available BET inhibitor, provided proof-of-concept for the activity of BET inhibition in NUT carcinoma.^22^ *MYC* is among the most frequently overexpressed oncogenes in human cancers, and preclinical studies have shown that BET inhibitors may also have activity in a broader range of tumor types, including mCRPC and CRC.^23^, ^24^, ^25^ Additionally, BET inhibition was shown to reduce SCLC cell proliferation via downregulation of *ASCL1* gene expression, providing rationale for the clinical development of BET inhibitors in this disease.^26^

Here, we report results from the phase Ia extension including DLBCL patients and phase Ib expansion solid tumor cohort.

## Methods

### Study design

The study design and findings from the initial phase Ia dose-escalation study have been published previously.^17^^,^^27^

The study design for this phase Ia/Ib open-label dose-escalation and dose-expansion study is summarized in Supplementary Figure S1, available at https://doi.org/10.1016/j.esmoop.2025.104499. The extension to the phase Ia dose escalation evaluated schedules B and C for patients with DLBCL. For patients with DLBCL, schedule B dosing started at 1.5 mg, one dose level lower than the MTD in solid tumors. schedule C started with a loading dose of BI 894999 4.0 mg on day 1 followed by 6 days of a lower maintenance dose (2.0 mg).

The phase Ib expansion cohorts evaluated schedule B 2.5 mg for patients with SCLC, mCRPC, CRC, and NUT carcinoma. schedule C, utilizing 6.0 mg/3.0 mg, was only evaluated in patients with NUT carcinoma.

The trial (Clinical Trial Registration: NCT02516553) was conducted and reported in accordance with the Declaration of Helsinki, the International Conference on Harmonisation—Good Clinical Practice guidelines, local regulations, and Boehringer Ingelheim standard operating procedures. All local ethics committees were informed and approved the trial. All patients provided written informed consent. Full details of the original protocol can be found in the Supplementary Data, available at https://doi.org/10.1016/j.esmoop.2025.104499.

The primary endpoint for the phase Ia DLBCL extension was the number of patients who experienced DLTs during the MTD evaluation period [first treatment cycle (3 weeks for schedule B; 4 weeks for schedule C)] to determine the MTD of both schedules.

The primary endpoint for the phase Ib expansion was the number of patients who experienced DLTs during the study treatment period.

Secondary endpoints for the phase Ia DLBCL extension and phase Ib expansion, including tumor response, progression-free survival (PFS), overall survival (OS) in patients with NUT carcinoma, adverse events (AEs), efficacy, pharmacokinetics (PK), and biomarkers of disease control, are described in more detail in Supplementary Table S1, available at https://doi.org/10.1016/j.esmoop.2025.104499.

### Patient eligibility

Eligible patients in the phase Ia DLBCL extension cohort had histologically confirmed DLBCL, had failed two or more lines of systemic therapy including an anti-CD20 therapy and an anthracycline, or were not amenable to standard therapies but had an indication for therapy per investigator judgment. Patients were >18 years old, had an Eastern Cooperative Oncology Group performance score (ECOG PS) of 0-2 at the time of screening, measurable disease according to Response Evaluation Criteria in Lymphoma (RECIL 2017) assessed via computerized tomography (CT), had recovered from therapy-related toxicities from previous anti-lymphoma therapy to Common Terminology Criteria for Adverse Events (CTCAE) grade ≤1 (with the exception of alopecia and grade 2 peripheral sensory neuropathy) and had a life expectancy of ≥12 weeks.

Eligible patients in phase Ib had histologically or cytologically confirmed diagnosis of an advanced unresectable and/or metastatic, malignant solid tumor from at least one of the four selected types (SCLC, mCRPC, CRC, NUT carcinoma) and had failed conventional treatment or for whom no therapy of proven efficacy existed and were not amenable to standard therapies. Patients were the legal adult age for the given country at the time of informed consent (except patients with NUT carcinoma who could be ≥15 years of age), had measurable disease and progressive disease within the past 6 months according to Response Evaluation Criteria in Solid Tumors (RECIST) 1.1 (except for patients with NUT carcinoma for whom only nonmeasurable disease was acceptable and who did not need to show progression) or according to Prostate Cancer Working Group 3 for patients with mCRPC. Patients had to have an accessible tumor lesion for biopsies (except for patients with mCRPC with only bone metastases or patients with therapeutic international normalized ratio due to treatment with a vitamin K antagonist or novel oral anticoagulant); biopsies were optional for patients with NUT carcinoma.

Specifically for the mCRPC cohort, patients had histologically or cytologically confirmed adenocarcinoma of the prostate, radiographic evidence of metastatic prostate cancer (TNM [tumour–node–metastasis] stage M1 or D2) where distant metastases were evaluable by bone scan, computerized tomography scan, or magnetic resonance imaging within 28 days before study start, had prostate specific antigen (PSA) ≥5 ng/ml (if no measurable disease by RECIST 1.1), had prior surgical or chemical castration with a serum testosterone of <50 ng/dl by luteinizing hormone-releasing hormone agonist or antagonist, or by abiraterone, enzalutamide, or apalutamide and had progressive disease defined as at least one of progressive measurable disease using RECIST 1.1; bone scan progression where at least two new lesions are observed on a bone scan plus a rising PSA; and an increasing PSA level where at least two consecutive PSA values rose above a reference value taken at least 1 week apart.

A comprehensive list of the eligibility criteria is provided in the protocol in the Supplementary Data, available at https://doi.org/10.1016/j.esmoop.2025.104499.

### Safety assessment

AEs were graded according to the CTCAE version 4.03. DLTs were defined as any treatment-related CTCAE grade ≥3 nonhematological toxicity (excluding inadequately treated nausea, vomiting, or diarrhea, and electrolyte abnormalities corrected within 72 hours of treatment), a CTCAE score of grade ≥4 neutropenia lasting ≥7 days and/or complicated by infection, CTCAE grade ≥4 thrombocytopenia, CTCAE grade ≥3 febrile neutropenia, or any other treatment-related AE preventing the patient from receiving treatment according to the given schedule (more than two consecutive doses missed). For fatigue at baseline, there had to be an increase of two or more grades.

The MTD was defined based on the frequency of patients experiencing DLTs during the MTD evaluation period, defined as the first treatment cycle with BI 894999 (the first 3 weeks for schedule B and the first 4 weeks for schedule C). The MTD was the highest dose with <25% risk of the true DLT rate being >33%.

### Efficacy assessment

PFS was measured for the period from the start of treatment to the occurrence of progressive disease or death. Tumor response was measured using RECIST 1.1^28^ for patients with solid tumors and RECIL 2017^29^ for those with DLBCL. OS was assessed in patients with NUT carcinoma; the OS status was to continue until 12 months after the end of the study.

### Pharmacokinetics

Samples were collected for PK analysis in cycle 1: 5 min pre-dose, 30 min, 1, 2, 3, 4, 6, and 8 h after drug administration on day 1 (schedules B and C) and on day 14 (for schedule B) or day 21 (for schedule C); 5 min pre-dose on days 8 and 12 (for schedule B) and at time of the visit on day 8 (168 h) for schedule C; 5 min pre-dose on day 18 (at time of the visit) for schedule B; and on day 22 (504 and 512 h) and day 29 (at time of the visit) for schedule C. For schedule B, a few additional samples were also taken in cycle 2: 5 min pre-dose, 2, 4, and 8 h on day 1, and 5 min pre-dose on day 2.

BI 894999 plasma concentrations were determined using a validated assay based on liquid chromatography coupled with tandem mass spectrometry. PK parameters were determined by noncompartmental analysis using the Phoenix WinNonlin software (Phoenix 6.3, Certara USA Inc., Princeton, NJ).

### Pharmacokinetic–pharmacodynamic relationship

The evaluation of biomarkers to identify patient subgroups with differential prognosis or response to treatment was exploratory, with no formal endpoints defined. Blood samples were assessed using an in-house-designed NanoString panel (NanoString, Seattle, WA) measuring gene expression levels of *HEXIM1*, *HIST2H2BF*, and *CCR2* over all time points, and postdose expression levels of *HEXIM1*, *HIST2H2BF*, and *CCR2* on days 14 and 15. *HEXIM1* is modulated by BET family proteins and is considered a robust pharmacodynamic marker of BET inhibition,^30^ whereas *HIST2H2BF* and *CCR2* are involved in cancer pathways.^31^^,^^32^ Target engagement was achieved if at least two of three of the genes demonstrated a twofold or more change induction (*HEXIM1* and *HIST2H2BF*) or suppression (*CCR2*) when compared with baseline.

### RNA isolation and analysis

Total RNA was extracted from whole blood using PAXgene Blood RNA kit (Qiagen, Hilden, Germany) following the manufacturer’s instructions. Total RNA was quantified by direct absorbance measurement at 260 nm using a NanoDrop Spectrophotometer (Thermo Fisher, Waltham, MA). Quality check was carried out by additional measurement of absorbance at 280 nm and calculation of the absorbance ratio OD260/OD280.

### NanoString analysis

mRNA expression was assessed using a custom nCounter® (NanoString, Seattle, WA) codeset generated for the genes of interest plus housekeeping genes. Probes were hybridized to 150 ng of total RNA for 19 h at 65°C and applied to the nCounter® Preparation Station for automated removal of excess probe and immobilization of probe–transcript complexes on a streptavidin-coated cartridge. Data were collected using the nCounter® Digital Analyzer by counting the individual barcodes. Data were analyzed using an in-house analysis pipeline that follows the latest NanoString Technologies standards for the analysis of nCounter® data. The analysis pipeline includes quality checking of the raw data using the NAnostring quality Control dasHbOard (NACHO), a comprehensive method to optimize the quality control of nCounter® data.^33^ The housekeeping genes *ACTB*, *GAPDH*, *HPRT1*, *MGAT1*, and *TMED9* were used for normalization.

### Molecular profiling

Tumor samples were sent to Foundation Medicine (Cambridge, MA) for molecular profiling. DNA was isolated and qualitative next-generation sequencing was undertaken for the detection of genomic alterations (substitutions, insertions, and deletions; copy number alterations; and select gene rearrangements). Genomic signatures to determine microsatellite status (MSS) and tumor mutational burden (TMB) were undertaken on the tissue sample.

### Statistical analysis

Dose escalation was guided by a Bayesian logistic regression model with overdose control fitted to binary toxicity outcomes.^34^ A Bayesian hierarchal model approach was used to analyze the response rate endpoints in phase Ib. Toxicity probability at each dose level was calculated to determine an estimate of the MTD. Exploratory analyses and descriptive statistics were used for secondary endpoints of toxicity.

## Results

### Patient demographics and oncological history

A total of 18 patients were enrolled in the phase Ia DLBCL extension and 79 patients in the phase Ib (Supplementary Figure S2, available at https://doi.org/10.1016/j.esmoop.2025.104499).

In the phase Ia extension, 14 patients with DLBCL were assigned to schedule B and four to schedule C. Most were male (77.8%) and aged >65 years (83.3%); 50.0% of patients were white and 55.6% had ECOG PS 0 (Table 1). In the phase Ib expansion, 57 patients were assigned to schedule B and 22 to schedule C. Most patients were male (69.6%), white (68.4%), and aged ≤65 years (68.4%); most had ECOG PS 1 (57.0%). Of the 79 patients in phase Ib, 42 (53.2%) had NUT carcinoma, 14 (17.7%) had CRC, 12 (15.2%) had SCLC, and 11 (13.9%) had mCRPC. Data regarding NUT carcinoma genomic and morphological features were not collected.

**Table 1:** Patient characteristics and oncological history

	DLBCL (phase Ia extension)			Solid tumor (phase Ib expansion)					
	Schedule B	Schedule C	Total	Schedule B				Schedule C	Total
				SCLC	CRC	mCRPC	NUT carcinoma	NUT carcinoma	
**Number of patients, *n***	14	4	18	12	14	11	20	22	79
**Male, *n* (%)**	11 (78.6)	3 (75.0)	14 (77.8)	7 (58.3)	8 (57.1)	11 (100)	15 (75.0)	14 (63.6)	55 (69.6)
**Median age, years (range)**	71.0 (58-87)	71.0 (67-86)	71.0 (58-87)	63.5 (53-77)	65.0 (52-77)	71.0 (63-76)	44.0 (22-71)	41.5 (20-75)	57.0 (20-77)
≤65, *n* (%)	3 (21.4)	0 (0.0)	3 (16.7)	7 (58.3)	8 (57.1)	2 (18.2)	18 (90.0)	19 (86.4)	54 (68.4)
>65 to ≤75, *n* (%)	5 (35.7)	3 (75.0)	8 (44.4)	4 (33.0)	5 (35.7)	8 (72.7)	2 (10.0)	3 (13.6)	22 (27.8)
>75, *n* (%)	6 (42.9)	1 (25.0)	7 (38.9)	1 (8.3)	1 (7.1)	1 (9.1)	0 (0.0)	0 (0.0)	3 (3.8)
**Race, *n* (%)**									
Asian	0 (0.0)	0 (0.0)	0 (0.0)	0 (0.0)	0 (0.0)	0 (0.0)	3 (15.0)	1 (4.5)	4 (5.1)
Black/African American	0 (0.0)	0 (0.0)	0 (0.0)	1 (8.3)	0 (0.0)	1 (9.1)	0 (0.0)	1 (4.5)	3 (3.8)
White	8 (57.1)	1 (25.0)	9 (50.0)	7 (58.3)	9 (64.3)	8 (72.7)	14 (70.0)	16 (72.7)	54 (68.4)
Missing	6 (42.9)	3 (75.0)	9 (50.0)	4 (33.3)	5 (35.7)	2 (18.2)	3 (15.0)	4 (18.2)	18 (22.8)
**ECOG performance score at baseline, *n* (%)**									
0	7 (50.0)	3 (75.0)	10 (55.6)	4 (33.3)	5 (35.7)	4 (36.4)	7 (35.0)	9 (40.9)	29 (36.7)
1	5 (35.7)	1 (25.0)	6 (33.3)	8 (66.7)	9 (64.3)	7 (63.6)	11 (55.0)	10 (45.5)	45 (57.0)
2	2 (14.3)	0 (0.0)	2 (11.1)	0 (0.0)	0 (0.0)	0 (0.0)	2 (10.0)	3 (13.6)	5 (6.3)
**TNM (tumour–node–metastasis) stage at diagnosis, *n* (%)**									
I	—	—	—	0 (0.0)	0 (0.0)	0 (0.0)	0 (0.0)	2 (9.1)	2 (2.5)
II	—	—	—	0 (0.0)	0 (0.0)	0 (0.0)	3 (15.0)	0 (0.0)	3 (3.8)
III	—	—	—	5 (41.7)	3 (21.4)	4 (36.4)	1 (5.0)	3 (13.6)	16 (20.3)
IV	—	—	—	7 (58.3)	11 (78.6)	7 (63.6)	16 (80.0)	15 (68.2)	56 (70.9)
Missing	14 (100.0)	4 (100.0)	18 (100.0)	0 (0.0)	0 (0.0)	0 (0.0)	0 (0.0)	2 (9.1)	2 (2.5)

CRC, colorectal cancer; DLBCL, diffuse large B-cell lymphoma; ECOG, Eastern Cooperative Oncology Group; mCRPC, metastatic castration-resistant prostate cancer; NUT, nuclear protein in testis; SCLC, small-cell lung cancer.

### Treatment exposure

In the phase Ia DLBCL extension, eight patients received BI 894999 1.5 mg, four received 2.0 mg, and two received 2.5 mg on schedule B. For schedule C, two patients received the 4.0 mg/2.0 mg dose and two received 5.0 mg/2.5 mg. Median treatment exposure was 35.5 days (range: 8-188 days). For most patients, either one [*n* = 6 (33.3%)] or two [*n* = 8 (44.4%)] treatment cycles were initiated. Three patients (16.7%) underwent dose reductions, with a median time to first dose reduction of 36.0 days (range: 36-71 days).

In the phase Ib expansion, 57 patients received BI 894999 schedule B (2.5 mg) and 22 received schedule C (6.0 mg/3.0 mg). Median treatment exposure was 44.0 days (range: 2-853 days). Most patients received one [*n* = 19 (24.1%)] or two treatment cycles [*n* = 29 (36.7%)]. Sixteen patients (20.3%) required a dose reduction, with median time to first dose reduction of 35.5 days (range: 2-755 days).

All 97 patients from the phase Ia DLBCL extension (*n* = 18) and phase Ib expansion (*n* = 79) discontinued BI 894999. Reasons for discontinuation included progressive disease [phase Ia DLBCL, *n* = 15 (83.3%); phase Ib, *n* = 62 (78.5%)], DLT [phase Ia DLBCL, *n* = 2 (11.1%); phase Ib, *n* = 3 (3.8%)], other AEs [phase Ia DLBCL, *n* = 1 (5.6%); phase Ib, *n* = 8 (10.1%)], refusal to continue medication [phase Ib, *n* = 2 (2.5%)], or other [phase Ib, *n* = 4 (5.1%)].

### Dose-limiting toxicities

Of the 18 patients enrolled on the phase Ia DLBCL extension, 17 were included in the MTD set (schedule B, *n* = 13; schedule C, *n* = 4) as one patient on schedule B was considered not assessable for DLT. Four patients on schedule B (30.8%; 1.5 mg, *n* = 1; 2.0 mg, *n* = 1; 2.5 mg, *n* = 2) had DLTs during the MTD evaluation period. No patients receiving schedule C experienced DLTs during the MTD period. DLTs during the MTD evaluation period included grade 4 thrombocytopenia (*n* = 3, 23.1%), grade 3 febrile neutropenia (*n* = 1, 7.7%), grade 4 sepsis (*n* = 1, 7.7%), and grade 3 increased troponin (*n* = 1, 7.7%) (Table 2). The MTD of schedule B was declared as 1.5 mg for patients with DLBCL. The MTD was not declared for schedule C. DLTs occurring during the on-treatment period in patients with DLBCL are shown in Supplementary Table S2, available at https://doi.org/10.1016/j.esmoop.2025.104499.

**Table 2:** Patients with DLBCL (phase Ia extension) and solid tumors (phase Ib expansion) experiencing grade ≥3 DLTs during the MTD evaluation and study treatment periods, respectively

DLBCL^a^	Dose	Patient reference number	DLT	Duration	Grade	Action	Therapy	Outcome	Serious
Phase Ia extension (DLBCL): total patients with DLTs on BI 894999 (schedule B; 1.5 mg, *n* = 1; 2.0 mg, *n* = 1; 2.5 mg, *n* = 2)									
	1.5 mg	#605	Increased troponin	5 days	3	None	No	Recovered	No
	2.0 mg	#130	Thrombocytopenia	11 days	4	Discontinued	No	Not recovered	No
	2.5 mg	#233	Thrombocytopenia	5 days	4	Reduced dose	Yes	Not recovered	No
		#402	Sepsis	6 days	4	Reduced dose	Yes	Recovered	Immediately life-threatening; required hospitalization
			Febrile neutropenia	7 days	3	Reduced dose	Yes	Recovered	Required hospitalization
			Thrombocytopenia	2 days	4	Reduced dose	Yes	Not recovered	No

Phase Ib expansion: total patients with DLTs on BI 894999 (schedule B; 2.5 mg), *n* = 14								
Tumor type	Patient reference number	DLT	Duration	Grade	Action	Therapy	Outcome	Serious
SCLC	#303	Laryngeal inflammation	—	3	Discontinued	Yes	Unknown	Required hospitalization
		Thrombocytopenia	—	4	None	Yes	Unknown	Required hospitalization
	#132	Thrombocytopenia	4 days	4	Discontinued	Yes	Not recovered	No
		Decreased appetite	9 days	3	None	No	Recovered	No
		Fatigue	—	3	None	No	Not recovered	No
		Troponin T increased	11 days	3	None	No	Recovered	No
	#505	Thrombocytopenia	6 days	4	None	Yes	Not recovered	Required hospitalization
CRC	#129	Dermatitis acneiform	—	3	None	Yes	Unknown	Required hospitalization
	#417	Thrombocytopenia	—	4	None	No	Not recovered	No
mCRPC	#230	Thrombocytopenia	40 days	4	Reduced dose	Yes	Not recovered	No
	#231	Thrombocytopenia	4 days	4	Reduced dose	No	Not recovered	No
	#308	Decreased platelet count	4 days	4	Reduced dose	Yes	Not recovered	Required hospitalization
	#403	Troponin T increased	—	3	None	No	Unknown	No
	#406	Thrombocytopenia	3 days	4	None	No	Not recovered	No
		Stomatitis	—	3	None	Yes	Not recovered	No
	#408	Nausea	—	3	Discontinued	Yes	Not recovered	Required hospitalization
		Decreased appetite	8 days	3	None	No	Recovered	No
		Hypophosphatemia	12 days	3	Reduced dose	Yes	Recovered	Required hospitalization
	#414	Thrombocytopenia	22 days	4	Discontinued	No	Not recovered	No
NUT carcinoma	#851	Decreased platelet count	—	4	Reduced dose	No	Not recovered	Required hospitalization
	#854	Decreased platelet count	6 days	4	None	No	Not recovered	Other

Phase Ib expansion: total patients with DLTs on BI 894999 (schedule C; 6.0/3.0 mg), *n* = 3								
NUT carcinoma	#1840007004	Pleural effusion	3 days	3	None	Yes	Recovered	Required hospitalization
		Fatigue	16 days	3	None	No	Not recovered	No
	#816	Fatigue	36 days	3	Reduced dose	No	Not recovered	No
		Diarrhea	3 days	3	Reduced dose	Yes	Not recovered	No
	#857	Decreased platelet count	—	4	None	Yes	Not recovered	Required hospitalization/prolonged hospitalization

CRC, colorectal cancer; DLBCL, diffuse large B-cell lymphoma; DLT, dose-limiting toxicity; mCRPC, metastatic castration-resistant prostate cancer; MTD, maximum tolerated dose; NUT, nuclear protein in testis; SCLC, small-cell lung cancer.

a No DLBCL patients on schedule C experienced DLTs on BI 894999.

In the phase Ib expansion, 17 patients (21.5%) had DLTs during the on-treatment period; 14 on schedule B (mCRPC, *n* = 7; SCLC, *n* = 3; CRC, *n* = 2; NUT carcinoma, *n* = 2) and three on schedule C (all with NUT carcinoma). The most frequently reported DLTs on treatment in the phase Ib expansion included grade 4 thrombocytopenia (*n* = 8, 10.1%), grade 4 decreased platelet count (*n* = 4, 5.1%), grade 3 fatigue (*n* = 3, 3.8%), grade 3 increased troponin T (*n* = 2, 2.5%), and grade 3 decreased appetite (*n* = 2, 2.5%) (Table 2).

### Safety

In the phase Ia DLBCL extension, 18 patients (100%) experienced AEs of any grade, with 15 patients (83.3%) determined as having treatment-related AEs (Table 3). The most common treatment-related AEs of any grade were thrombocytopenia (61.1%), diarrhea (50.0%), nausea (27.8%), anemia (27.8%), neutropenia (22.2%), fatigue (22.2%), decreased platelet count (11.1%), and increased troponin (11.1%). The most common grade ≥3 treatment-related AEs were thrombocytopenia (44.4%), anemia (27.8%), neutropenia (16.7%), and decreased platelet count (11.1%). Dose reductions and dose discontinuations due to AEs occurred in three (16.7%) and four (22.2%) patients, respectively. AEs leading to dose discontinuations included thrombocytopenia (11.1%), lymph node pain (5.6%), and neutropenia (5.6%).

**Table 3:** Overall summary of AEs

	DLBCL (phase Ia extension) *n* = 18	Solid tumors (phase Ib expansion) *n* = 79
Any AE, *n* (%)	18 (100)	79 (100)
Treatment-related AEs, *n* (%)	15 (83.3)	66 (83.5)
Treatment-related serious AEs, *n* (%)	2 (11.1)	17 (21.5)
AEs leading to dose reduction, *n* (%)	3 (16.7)	13 (16.5)
AEs leading to discontinuation of trial drug, *n* (%)	4 (22.2)	11 (13.9)
DLT in on-treatment period, *n* (%)	8 (44.4)	17 (21.5)
AEs leading to death, *n* (%)	1 (5.6)	7 (8.9)

DLBCL (phase Ia extension)		
Treatment-related AEs occurring in ≥10% of patients, *n* (%)	Any grade	Grade ≥3
Thrombocytopenia	11 (61.1)	8 (44.4)
Diarrhea	9 (50.0)	0
Nausea	5 (27.8)	0
Anemia	5 (27.8)	5 (27.8)
Neutropenia	4 (22.2)	3 (16.7)
Decreased appetite	4 (22.2)	0
Fatigue	4 (22.2)	0
Decreased platelet count	2 (11.1)	2 (11.1)
Troponin increased	2 (11.1)	1 (5.6)
Dysgeusia	2 (11.1)	0
Hematoma	2 (11.1)	0
Epistaxis	2 (11.1)	0
Muscle spasms	2 (11.1)	0
Asthenia	2 (11.1)	0

Solid tumors (phase Ib expansion)								
	SCLC *n* = 12		CRC *n* = 14		mCRPC *n* = 11		NUT carcinoma total *n* = 42	
**Number of patients with treatment-related AEs, *n* (%)**	11 (91.7)		12 (85.7)		11 (100.0)		32 (76.2)	

Treatment-related AEs occurring in ≥10% of patients, *n* (%)	Any grade	Grade ≥3	Any grade	Grade ≥3	Any grade	Grade ≥3	Any grade	Grade ≥3

Thrombocytopenia	5 (41.7)	3 (25.0)	5 (35.7)	3 (21.4)	5 (45.5)	4 (36.4)	10 (23.8)	6 (14.3)
Fatigue	4 (33.3)	1 (8.3)	4 (28.6)	0	4 (36.4)	0	10 (23.8)	4 (9.5)
Diarrhea	3 (25.0)	0	6 (42.9)	2 (14.3)	6 (54.5)	0	8 (19.0)	1 (2.4)
Dysgeusia	3 (25.0)	0	3 (21.4)	0	4 (36.4)	0	5 (11.9)	0
Anemia	0	0	3 (21.4)	2 (14.3)	4 (36.4)	4 (36.4)	11 (26.2)	6 (14.3)
Decreased appetite	2 (16.7)	1 (8.3)	5 (35.7)	0	4 (36.4)	1 (9.1)	1 (2.4)	0
Vomiting	2 (16.7)	0	3 (21.4)	2 (14.3)	5 (45.5)	0	0	0
Nausea	1 (8.3)	0	3 (21.4)	0	4 (36.4)	1 (9.1)	2 (4.8)	0
Weight decrease	1 (8.3)	0	1 (7.1)	0	2 (18.2)	0	0	0
Epistaxis	0	0	1 (7.1)	0	2 (18.2)	0	1 (2.4)	0
ALT increase	1 (8.3)	0	0	0	0	0	5 (11.9)	0
Decreased platelet count	1 (8.3)	0	1 (7.1)	1 (7.1)	3 (27.3)	1 (9.1)	15 (35.7)	7 (16.7)

AE, adverse event; ALT, alanine aminotransferase; CRC, colorectal cancer; DLBCL, diffuse large B-cell lymphoma; DLT, dose-limiting toxicity; mCRPC, metastatic castration-resistant prostate cancer; NUT, nuclear protein in testis; SCLC, small-cell lung cancer.

In the phase Ib expansion cohort, 79 patients (100%) experienced AEs of any grade, with 66 patients (83.5%) with treatment-related AEs (Table 3). The most common treatment-related AEs were thrombocytopenia (31.6%), diarrhea (29.1%), fatigue (27.8%), decreased platelet count (25.3%), anemia (22.8%), dysgeusia (19.0%), decreased appetite (15.2%), vomiting (12.7%), and nausea (12.7%). The most common grade ≥3 treatment-related AEs were thrombocytopenia (20.3%), anemia (15.2%), decreased platelet count (11.4%), and fatigue (6.3%). Dose reductions and dose continuations due to AEs occurred in 13 (16.5%) and 11 (13.9%) patients, respectively. AEs leading to dose discontinuations included thrombocytopenia (2.5%), tumor hemorrhage, seizure, dyspnea, laryngeal inflammation, nausea, stomatitis, bladder dilation, urinary retention, increased alanine aminotransferase, increased aspartate aminotransferase, decreased platelet count, and increased alkaline phosphatase (each 1.3%). Seven patients (8.9%) died because of AEs, including coronavirus disease (COVID-19), pneumonia, sepsis, delirium, seizure, dyspnea, pulmonary hemorrhage, and acute kidney injury (each 1.3%).

### Efficacy

In the phase Ia DLBCL extension (*n* = 18), the BOR was a confirmed PR [duration of response (DOR): 49 days] in one patient (5.6%) (Supplementary Figure S3 and Supplementary Table S3, available at https://doi.org/10.1016/j.esmoop.2025.104499). Three patients (16.7%) had SD, nine patients (50.0%) had progressive disease, and five patients (27.8%) were not assessable.

In the phase Ib expansion (*n* = 79), the BOR was a confirmed CR in one patient (1.3%) with NUT carcinoma (schedule C; Supplementary Figure S3 and Supplementary Table S3, available at https://doi.org/10.1016/j.esmoop.2025.104499). Among 42 patients with NUT carcinoma, 16 patients had disease control, including one patient (2.4%) with a confirmed CR (DOR: 253 days), one patient (2.4%) with confirmed PR (DOR: 588 days), one patient (2.4%) with an unconfirmed PR (DOR not available due to patient refusal to continue the trial after first imaging), and 13 patients (31.0%) with SD. Eighteen patients (42.9%) had progressive disease and eight patients (19.0%) were not assessable. Responses in other solid tumor types (*n* = 37) included one patient (2.7%) with mCRPC who had a confirmed PR (DOR: 46 days) and five patients (13.5%) with SD (SCLC, *n* = 1; CRC, *n* = 2; mCRPC, *n* = 2). Twenty-two patients (59.5%) had progressive disease, and nine patients (24.3%) were not assessable.

Median PFS was 6.9 weeks [95% confidence interval (CI) 6.0-8.1 weeks] in phase Ib (Supplementary Table S3, available at https://doi.org/10.1016/j.esmoop.2025.104499). Patients with mCRPC had the longest median PFS of 11.9 weeks (95% CI 8.1-24.4 weeks), whereas patients with SCLC and CRC had the shortest median PFS of 5.6 weeks (95% CI 5.3-6.1 weeks and 4.4-10.7 weeks, respectively). Patients with NUT carcinoma had a median PFS of 6.9 weeks (95% CI 6.0-11.1 weeks) and 7.8 weeks (95% CI 4.0-13.0 weeks) on schedules B and C, respectively.

Median OS in patients with NUT carcinoma was 6.6 weeks (95% CI 6.6 weeks to not estimable) for patients receiving schedule B and 15.4 weeks (95% CI 7.6-32.3 weeks) for schedule C (Supplementary Table S3, available at https://doi.org/10.1016/j.esmoop.2025.104499).

### Pharmacokinetics

**Figure 1:**
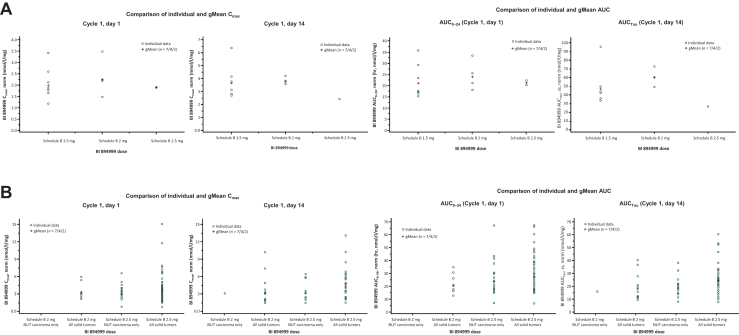
Pharmacokinetic parameters showing maximal exposure and area under the plasma concentration–time curve over the 24-h dosing interval for (A) DLBCL (phase Ia extension) and (B) solid tumor (phase Ib expansion). Top left: Cmax, maximal exposure; gMean, geometric mean; Top right: gMean, geometric mean; AUC0–24; area under the plasma concentration–time curve over the 24-h dosing interval; AUCtau, area under the plasma concentration–time curve over the dosing interval tau; Bottom left: Cmax, maximal exposure; gMean, geometric mean; NUT, nuclear protein in testis; Bottom right: AUC0–24, area under the plasma concentration–time curve over the 24-h dosing interval; AUCtau, area under the plasma concentration–time curve over the dosing interval tau; gMean, geometric mean; NUT, nuclear protein in testis.

Pharmacokinetics was evaluable for 97 patients in the phase Ia DLBCL extension and phase Ib solid tumor expansion. Median time to maximum plasma concentration (t_max_) was observed ∼2 h after single-dose administration at day 1 and after once-daily multiple doses at days 14 or 21 (Figure 1).

BI 894999 plasma concentrations increased with increasing dose within a dosing schedule and indication. For the 6.0 mg/3.0 mg (schedule C), comparison of pharmacokinetics parameters of patients with NUT carcinoma versus solid tumors resulted in similar geometric mean (gMean) plasma peaks and exposure following both single and multiple dosing. This was also observed for patients with DLBCL versus those with solid tumors (2 mg and 2.5 mg doses, schedule B) (Supplementary Table S4, available at https://doi.org/10.1016/j.esmoop.2025.104499).

### Pharmacodynamics

**Figure 2:**
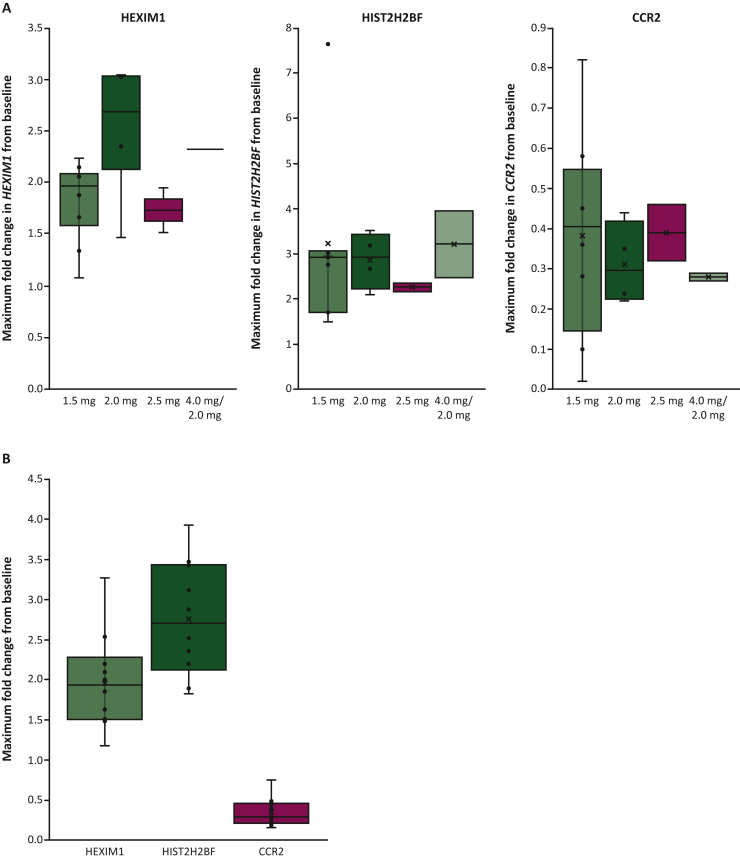
Maximum fold change in *HEXIM1*, *HIST2H2BF*, and *CCR2* expression from baseline in patients with (A) DLBCL (phase Ia extension) and (B) NUT carcinoma (phase Ib expansion). DLBCL, diffuse large B-cell lymphoma; NUT, nuclear protein in testis.

Pharmacodynamic profiles were available for 16 patients with DLBCL for the phase Ia extension and 41 patients with solid tumors in the phase Ib expansion. In the phase Ia DLBCL expansion, the median maximum fold change in gene expression from baseline superseded the predesignated twofold or greater change from baseline for both *HIST2H2BF* and *CCR2*. *HEXIM1* also superseded twofold or greater change from baseline at 2.0 mg but not at 1.5 mg or 2.5 mg (Figure 2). Since the target engagement was considered achieved if at least two of three genes demonstrated a twofold or greater change induction (*HEXIM1* and *HIST2H2BF*) or suppression (*CCR2*) compared with baseline, target engagement was seen at all dose levels in phase Ia DLBCL patient blood samples.

In the phase Ib extension, blood samples from the various solid tumor entities all showed target engagement based on the above designated criteria. Only in NUT carcinoma was *HEXIM1* induction marginally below the predesignated twofold or greater change (median fold change of 1.93) from baseline (Figure 2; Supplementary Figure S4, available at https://doi.org/10.1016/j.esmoop.2025.104499). Target engagement was achieved as two of the three genes demonstrated a twofold or greater change induction (*HEXIM1* and *HIST2H2BF*) or suppression (*CCR2*) compared with baseline.

### Molecular profiling

Molecular profiling was undertaken for seven patients with CRC. The exon coverage ranged from 471 to 852. Known somatic short variants were detected in a variety of genes (Supplementary Table S5, available at https://doi.org/10.1016/j.esmoop.2025.104499). Other identified genomic alterations are also listed. All samples were classified as MSS and low/intermediate TMB status (3.51-11.41 mutations/mb).

## Discussion

Here we report the final results from a phase Ia/Ib study evaluating BI 894999 in patients with DLBCL and those with SCLC, CRC, mCRPC, or NUT carcinoma. DLTs of interest were thrombocytopenia and increased troponin T. This was in line with previous results for the phase Ia part of the study.^17^ No new safety signals were identified, with the most frequently reported treatment-related AEs being thrombocytopenia, anemia, fatigue, and diarrhea.

Some patients experienced elevated troponin T levels, leading to a review of troponin T levels, electrocardiogram data, clinical summaries, and non-Good Laboratory Practice preclinical assays. The increased troponin T did not correlate with clinical or electrocardiogram changes. There was no preclinical evidence of potential cardiac toxicity related to BI 894999, or a BI 894999-related troponin T increase. This also corresponded with a lack of cardiotoxicity reported in preclinical studies in animals^17^ and this was not a toxicity previously reported with BET inhibitors.^35^ It was concluded that the cause of troponin T elevation may be multifactorial, but not clinically significant to preclude further clinical development.^17^

Across all 174 patients in the Ia and Ib stages of the study, including patients reported previously in phase Ia,^17^ the best response achieved was a CR in one patient of the 42 patients with NUT carcinoma, with an additional seven patients achieving a PR. As a result, development of BI 894999 was terminated as the observed objective response rate of <10% in NUT carcinoma did not support further recruitment. In addition, the clinical efficacy of BI 894999 in patients with DLBCL on schedule B in the phase Ia dose expansion was insufficient to warrant further development in DLBCL.

Given the low likelihood for BI 894999 monotherapy to confer a significant benefit in patients with NUT carcinoma or other solid tumors, no further development will be pursued. There have been mixed results for BET inhibitors in clinical trials, with most recent studies reporting only moderate efficacy and burdensome safety profiles.^18^^,^^36^, ^37^, ^38^ Considering the limited effect of BET inhibitor monotherapy, rational combinations, such as with chemotherapy, tyrosine kinase inhibitors, cyclin-dependent kinase inhibitors, inhibitors of cyclic AMP response binding protein-binding protein/p300, proteasome inhibitors or immunotherapy, may be considered to improve efficacy.^13^^,^^38^^,^^39^ Preclinical studies have demonstrated the synergistic antitumor effects of BET inhibitor combinations, such as in ovarian cancer, DLBCL, and CRC cell lines and SCLC xenograft models.^2^^,^^40^, ^41^, ^42^ Numerous phase I/II clinical studies are in progress investigating different BET inhibitor combination regimens in multiple cancer types.^43^ Of note, a phase Ib trial of BET inhibitor RO6870810 in combination with venetoclax and rituximab in 39 patients with relapsed/refractory DLBCL revealed an overall response rate of 38.5% and complete responses in 20.5% of patients.^44^ Similarly, a phase I study of BET inhibitor mivebresib alone or in combination with venetoclax in 44 patients with relapsed/refractory acute myeloid leukemia revealed higher efficacy in the combination group compared with mivebresib monotherapy.^45^

### Conclusions

In this phase Ia/b study, the safety profile of BI 894999 was in line with that reported for other BET inhibitors. Although there were objective responses in patients with NUT carcinomas, including one CR, BI 894999 did not demonstrate sufficient efficacy to warrant further investigation.

## Disclosure

**UML**: research funding and grants or contracts for Boehringer Ingelheim; consultancy fees for Boehringer Ingelheim and Abalos Therapeutics, and advisory boards for Asgard Therapeutics. **AA**: advisory and speaker fees from Amgen, AstraZeneca, Bayer, Daiichi, Eisai, Genomic Health, Ipsen, LEO Pharma, Lilly, Merck, Merck Sharp & Dohme (MSD), Novartis, Pfizer, and Seattle Genetics; advisory fees received from Hengrui, Innate, and Viatris; and research grants received from Bristol Myers Squibb (BMS) and Roche. **SPV**: principal/sub-investigator of clinical trials for AbbVie, Adaptimmune, Adlai Nortye USA, Inc., Aduro Biotech, Agios Pharmaceuticals, Amgen, Astex Pharmaceuticals, AstraZeneca AB, Aveo, Basilea Pharmaceutica International Ltd, Bayer Healthcare AG, BBB Technologies BV, Beigene, BicycleTx Ltd, Blueprint Medicines, Boehringer Ingelheim, Boston Pharmaceuticals, BMS, Ca, Casi Pharmaceuticals, Inc., Celgene Corporation, Cellcentric, Chugai Pharmaceutical Co, Cullinan-Apollo, CureVac, Daiichi Sankyo, Debiopharm, Eisai, Eisai Limited, Eli Lilly, Exelixis, Faron Pharmaceuticals Ltd, Forma Therapeutics, Gamamabs, Genentech, GSK, H3 Biomedicine, Hoffmann-La Roche AG, Imcheck Therapeutics, Incyte Corporation, Innate Pharma, Institut de Recherche Pierre Fabre, Iris Servier, Iteos Belgium SA, Janssen Cilag, Janssen Research Foundation, Janssen R&D LLC, Kura Oncology, Kyowa Kirin Pharmaceutical Development, Lilly France, Loxo Oncology, Medimmune, Menarini Ricerche, MSD Chibret, Merrimack Pharmaceuticals, Merus, Molecular Partners AG, Nanobiotix, Nektar Therapeutics, Novartis Pharma, Octimet Oncology NV, Oncoethix, Oncopeptides, Orion Pharma, Genomics, Ose Pharma, Pfizer, Pharma Mar, Pierre Fabre Medicament, Relay Therapeutics, Inc., Roche, Sanofi Aventis, Seattle Genetics, Sotio A.S, Syros Pharmaceuticals, Taiho Pharma, Tesaro, Transgene S.A, Turning Point Therapeutics, Xencor; research grants received from Roche, AstraZeneca; and nonfinancial support (drug supplied) from Roche, Clovis, and GSK. **GIS**: grants from Pfizer, Eli Lilly, Merck KGaA/EMD Serono, Tango, BMS, and Merck & Co; and personal fees from Bicycle Therapeutics, Cybrexa Therapeutics, Bayer, Boehringer Ingelheim, ImmunoMet, Artios, Concarlo Holdings, Syros, Zentalis, CytomX Therapeutics, Blueprint Medicines, Kymera Therapeutics, Janssen, and Xinthera. **CT**: institutional research funding from Kite/Gilead and Roche; honoraria for advisory boards from Roche, Novartis, AstraZeneca, BeiGene, AbbVie, Takeda, Kite/Gilead, and BMS; and travel and accommodation expenses from Roche, Novartis, AbbVie, Takeda, Roche, Kite/Gilead, and BMS. **SAPP**: clinical trial research support/grant funding through the institution from AbbVie, Inc., ABM Therapeutics, Inc., Acepodia, Inc., Alkermes, Aminex Therapeutics, Amphivena Therapeutics, Inc., BioMarin Pharmaceutical, Inc., Boehringer Ingelheim, BMS, Cerulean Pharma, Inc., Chugai Pharmaceutical Co., Ltd., Curis, Inc., Cyclacel Pharmaceuticals, Daiichi Sankyo, Eli Lilly, ENB Therapeutics, Epigenetix Inc., Five Prime Therapeutics, FStar Beta Limited, F-Star Therapeutics, Gene Quantum, Genmab A/S, Gilead Sciences, Inc., GSK, Helix BioPharma Corp., Hengrui Pharmaceuticals, Co., Ltd., HiberCell, Inc., Immorna Biotherapeutics, Inc., Immunomedics, Inc., Incyte Corp., Jacobio Pharmaceuticals Co., Ltd., Jiangsu Simcere Pharmaceutical Co., Ltd., Lytix Biopharma AS, Medimmune, LLC., Medivation, Inc., MSD Corp., Nectin Therapeutics, Ltd., Novartis Pharmaceuticals, Pieris Pharmaceuticals, Inc., Pfizer, Phanes Therapeutics, Principia Biopharma, Inc., Puma Biotechnology, Inc., Purinomia Biotech, Inc., Rapt Therapeutics, Inc., Replimune, Seattle Genetics, Silverback Therapeutics, Synlogic Therapeutics, Taiho Oncology, Tesaro, Inc., TransThera Bio, ZielBio, Inc., NCI/NIH, P30CA016672dCore Grant (CCSG Shared Resources); and consulting fees from CRC Oncology. **PKP**: consulting or advisory board fees from Bicara Therapeutics, EMD Serono, Takeda Pharmaceuticals, Janssen, Mirati, and Novartis; honoraria for participation in CME educational programs from PeerVoice, ACE Oncology, IDEOlogy, Physicians Education Resource, Medscape, Aptitude Health, AXIS Medical Education, and Touch Independent Medical Education; institutional research grants from EMD Serono and Bicara. **DRS**: honoraria for Deciphera; consulting or advisory roles for AADi, Deciphera, Replimune, and Sanofi; research funding (institution) for Amgen, Astellas Pharma, AstraZeneca, Bayer, Boehringer Ingelheim, BMS, Compugen, Genentech/Roche, Harpoon Therapeutics, InhibRx, Lilly, MabSpace Biosciences, Novartis, Pionyr, Shanghai Miracogen Inc., Tempus, and VBL Therapeutics. **LID**: payment or honoraria for lectures, presentations, speaker bureaus, manuscript writing, or educational events from Merck, MSD, Roche, Bayer, and Esai; support for attending meetings and/or travel from Merck and MSD; participated in data safety monitoring or advisory board for Merck, MSD, Roche, Lilly, and Ipsen. **RG**: travel, accommodation, and expenses from Merck. **SR**: travel grants and advisory/speaker fees from Novartis, Roche, Pfizer, MSD, BMS, Ipsen; research grants from Roche and MSD. **BS** and **KM**: employees of Boehringer Ingelheim. **HM**: former employee of Boehringer Ingelheim. **PS**: consulting or advisory role for Deciphera, Ellipses Pharma, Transgene, Exelixis, Boehringer Ingelheim, Studiecentrum voor Kernenergie, SQZ Biotechnology, Adcendo, PharmaMar, Merck Healthcare KGaA, Medpace, Cogent Biosciences, Eisai, Curio Science, LLX Solutions, SERVIER, Genmab, Biolumina, Sanofi, Regeneron, Moleculin Biotech, Avacta Life Sciences, Amryt Pharma, UCB, Boxer Capital; and research funding for CoBioRes NV, Eisai, G1 Therapeutics, PharmaMar, Genmab, Merck, Sartar Therapeutics, ONA Therapeutics, and Adcendo.
