# Supplementary material for: Final results from the phase Ia/Ib study of the novel bromodomain and extra-terminal domain inhibitor, BI 894999, in patients with advanced solid tumors or diffuse large B-cell lymphoma
Source: ESMO Open. 2025 Apr 8;10(5):104499. doi: 10.1016/j.esmoop.2025.104499 (PMC12005229; doi:10.1016/j.esmoop.2025.104499)
Supplement: Supplementary Material [file mmc1.pdf]

Supplementary material

Supplementary Figure 1. Phase Ia/Ib study design in (A) patients with solid tumors and in (B) patients with DLBCL

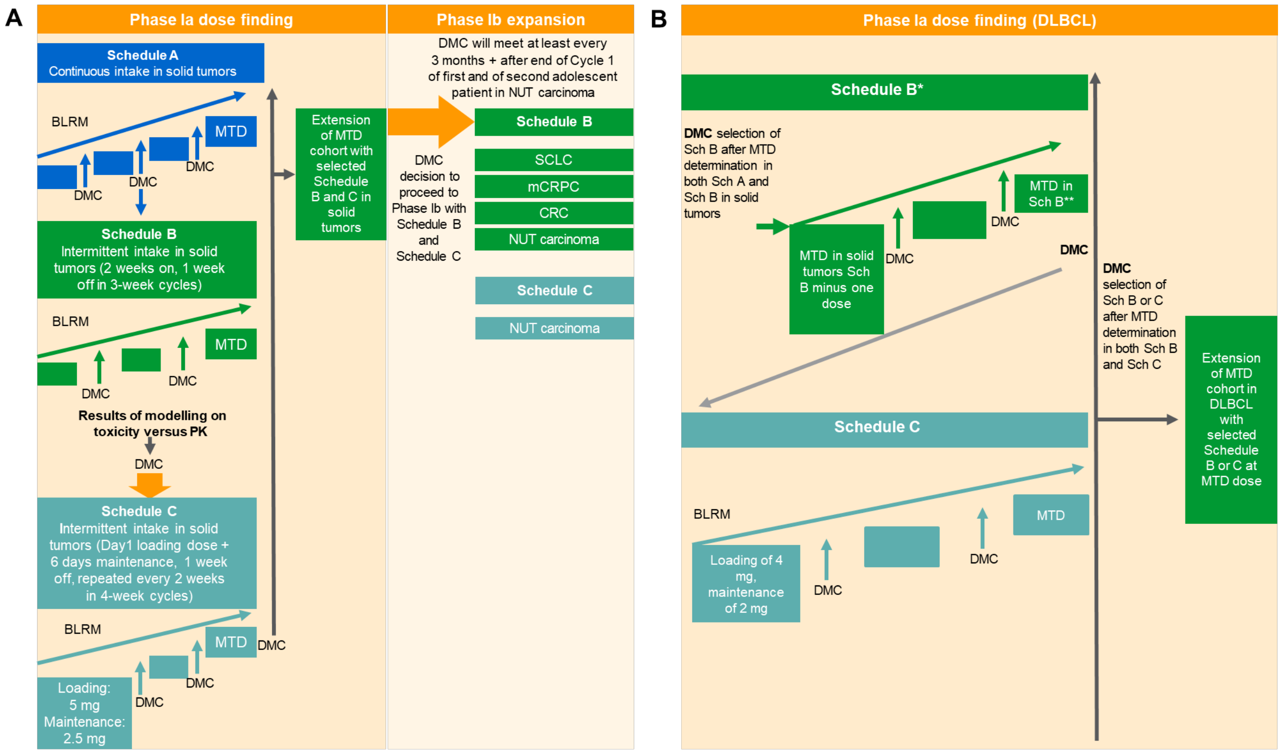

BLRM, Bayesian logistic regression model; CRC, colorectal cancer; DLBCL, diffuse large B-cell lymphoma; DMC, Data Monitoring Committee; mCRPC, metastatic castration-resistant prostate cancer; MTD, maximum tolerated dose; NUT, nuclear protein in testis; PK, pharmacokinetics; SCLC, small-cell lung cancer.

Supplementary Figure 2. Patient disposition

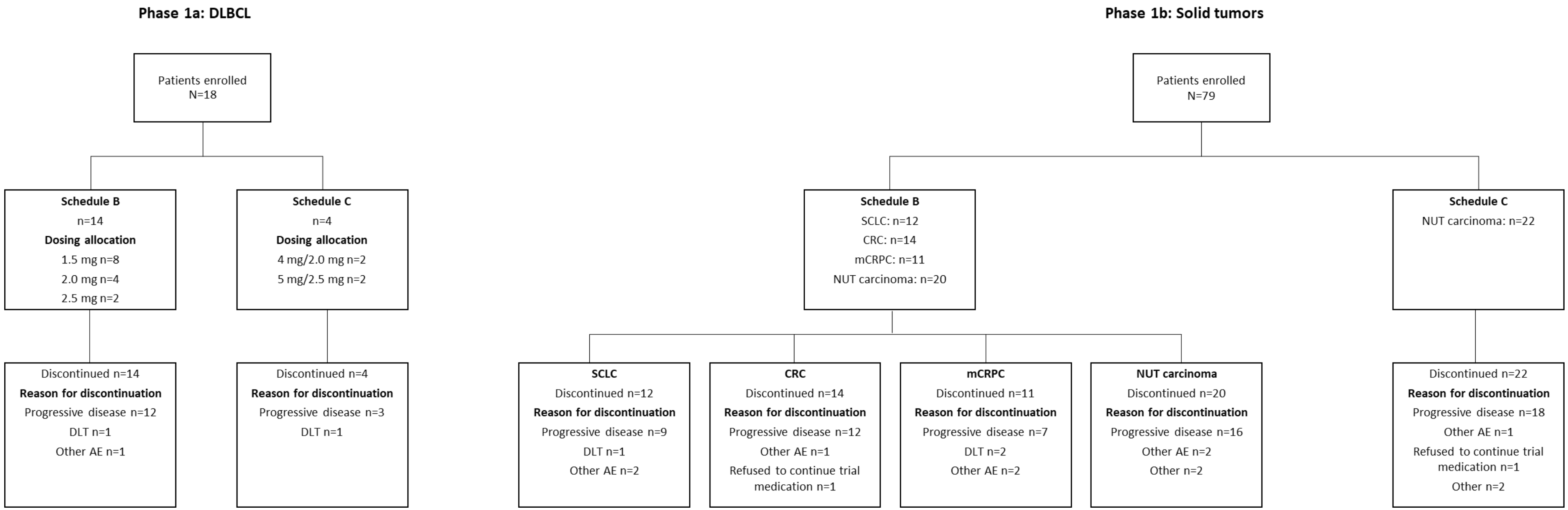

AE, adverse event; CRC, colorectal cancer; DLBCL, diffuse large B-cell lymphoma; DLT, dose-limiting toxicity; mCRPC, metastatic castration-resistant prostate cancer; NUT, nuclear protein in testis; SCLC, small-cell lung cancer.

Supplementary Figure 3. Waterfall plot of best percentage change from baseline in lesion measurement for (A) patients with DLBCL (Phase Ia extension), (B) all patients with solid tumors (Phase Ib expansion) and (C) Phase Ib NUT carcinoma only

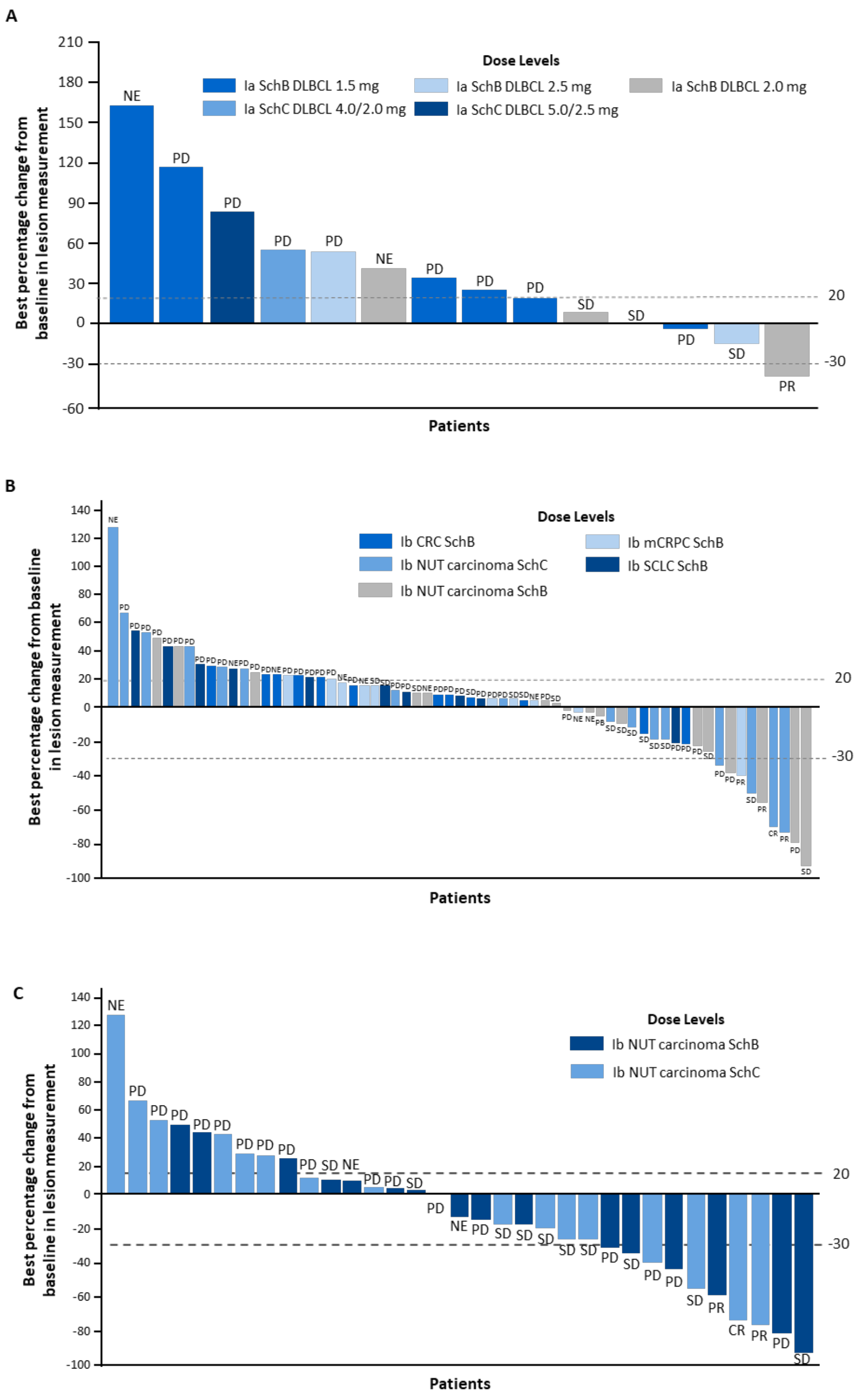

Note: Patients with missing measurements are not included in waterfall plot.

CR, complete response; DLBCL, diffuse large B-cell lymphoma; NUT, nuclear protein in testis; NE, not evaluable; PD, progressive disease; PR, partial response; SD, stable disease.

Supplementary Figure 4. Median maximum fold change in HEXIM1, HIST2H2BF and CCR2 expression from baseline in patients in the Phase 1b expansion with (A) SCLC, (B) CRC and (C) mCRPC

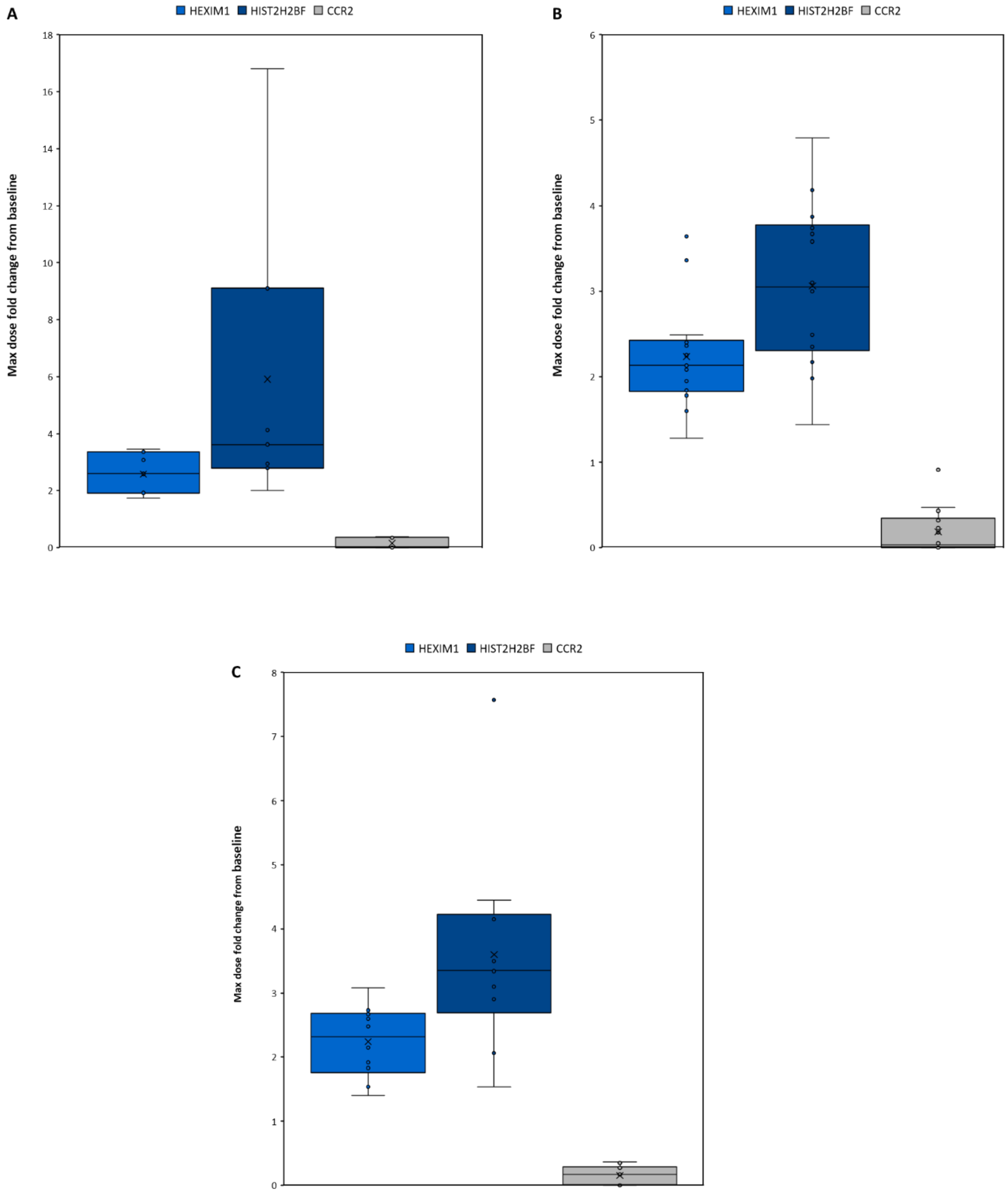

CRC, colorectal cancer; mCRPC, metastatic castration-resistant prostate cancer; SCLC, small-cell lung cancer.

Supplementary Table 1. Secondary efficacy and pharmacokinetic endpoints

**Phase Ia and Ib**
• Pharmacokinetic parameters after single dose and at steady state, measured during the first cycle (the first 3 weeks for Schedule B, the first 4 weeks for Schedule C)<br>○ C_{max}<br>○ $AUC_{0\text{-}24}$<br>○ $C_{max,ss}$<br>○ $AUC_{\tau,ss}$<br>• Objective response (OR), defined as complete response (CR) or partial response (PR) with tumor assessment during treatment period for each schedule according to Response Evaluation Criteria in Solid Tumors (RECIST) version 1.1. For DLBCL patients, a minor response according to Response Evaluation Criteria in Lymphoma (RECIL) 2017 was not part of an OR
**Phase Ib**
• Progression-free survival (PFS), defined from date of start of BI 894999 to the date of objective disease progression or death, whichever was earlier, with tumor assessment every 2 cycles (every 6 weeks for Schedule B if no delays or as close as possible to the end of the second of the 2 cycles of treatment if there was a delay) during the treatment period<br>OR<br>Radiological PFS with tumor assessment by bone scan every 4 cycles for mCRPC patients with non-measurable disease by RECIST 1.1<br>• Best overall response, with tumor assessment, depending on the type of solid tumor, during the treatment period<br>• Prostate serum antigen response in patients with mCRPC<br>• Overall survival in patients with NUT carcinoma. This was for patients in the trial after approval of protocol version 11.0 and who gave consent to this data collection. The overall survival status was to continue until 12 months after the end of trial
**Secondary safety endpoints**
**Phase Ia**
• The number of patients with DLTs observed during all treatment cycles for Schedules B and C in the DLBCL cohort<br>• All DLTs occurring during the first or repeated treatment cycles were to be reported as significant AEs, as for a serious adverse event

$AUC_{0–24}$, area under the plasma concentration–time curve over the 24-hour dosing interval; $AUC_{\tau,ss}$, area under the plasma concentration–time curve over the dosing interval τ at steady state; AE, adverse event; C_{max}, maximal exposure; $C_{max,ss}$, maximal exposure at steady state; DLBCL, diffuse large B-cell lymphoma; DLT, dose-limiting toxicity; mCRPC, metastatic castration-resistant prostate cancer; NUT, nuclear protein in testis.

Supplementary Table 2. Phase Ia DLBCL: Patients with DLTs Grade ≥3 during the on-treatment period

**Schedule B: Total patients with DLTs on BI 894999 during the on-treatment period, n=5**								
**Dose, mg**	**Patient no.**	**DLT**	**Duration, days**	**Grade**	**Action**	**Therapy**	**Outcome**	**Serious**
1.5	#605	Increased troponin	5	3	None	No	Recovered	No
2.0	#130	Thrombocytopenia	11	4	Discontinued	No	Not recovered	No
	#229	Melena	7	3	None	No	Recovered	No
2.5	#233	Thrombocytopenia	5	4	Reduced dose	Yes	Not recovered	No
	#402	Sepsis	6	4	Reduced dose	Yes	Recovered	Immediately life threatening; required hospitalization
		Febrile neutropenia	7	3	Reduced dose	Yes	Recovered	Required hospitalization
		Thrombocytopenia	2	4	Reduced dose	Yes	Not recovered	No
**Schedule C: Total patients with DLTs on BI 894999 during the on-treatment period, n=3**								
**Dose, mg**	**Patient no.**	**DLT**	**Duration, days**	**Grade**	**Action**	**Therapy**	**Outcome**	**Serious**
4.0/2.0	#422	Decreased platelet count	-	4	None	No	Not recovered	No
5.0/2.5	#1250004001	Neutropenia	-	4	Discontinued	No	Not recovered	No
	#610	Thrombocytopenia	8	4	None	No	Not recovered	No

DLBCL, diffuse large B-cell lymphoma; DLT, dose-limiting toxicity.

Supplementary Table 3. Tumor response[a] for Phase Ia DLBCL/Ib efficacy

	Phase Ia DLBCL		
	**Schedule B**	**Schedule C**	**Total**
Patients, n (%)	14 (100.0)	4 (100.0)	18 (100.0)
Best overall response, n (%)			
Complete response	0 (0.0)	0 (0.0)	0 (0.0)
Partial response	1 (7.1)	0 (0.0)	1 (5.6)
Stable disease	2 (14.3)	1 (25.0)	3 (16.7)
Progressive disease	6 (42.9)	3 (75.0)	9 (50.0)
Not evaluable	5 (35.7)	0 (0.0)	5 (27.8)
Objective response[b], n (%)	1 (7.1)	4 (100.0)	1 (5.6)
Disease control[c], n (%)	3 (21.4)	1 (25.0)	4 (22.2)

	**Phase Ib**					
	**SCLC**	**CRC**	**mCRPC**	**NUT carcinoma (Schedule B)**	**NUT carcinoma (Schedule C)**	**Total**
Patients, n (%)	12 (100.0)	14 (100.0)	11 (100.0)	20 (100.0)	22 (100.0)	79 (100.0)
Best overall response, n (%)						
Complete response	0 (0.0)	0 (0.0)	0 (0.0)	0 (0.0)	1 (4.5)	1 (1.3)
Partial response	0 (0.0)	0 (0.0)	1 (9.1)	1 (5.0)	1 (4.5)	3 (3.8)
Stable disease	1 (8.3)	2 (14.3)	2 (18.2)	7 (35.0)	6 (27.3)	18 (22.8)
Progressive disease	8 (66.7)	10 (71.4)	4 (36.4)	9 (45.0)	9 (40.9)	40 (50.6)
Not evaluable	3 (25.0)	2 (14.3)	4 (36.4)	3 (15.0)	5 (22.7)	17 (21.5)
Objective response[b], n (%)	0 (0.0)	0 (0.0)	1 (9.1)	1 (5.0)	2 (9.1)	4 (5.1)
Disease control[c], n (%)	1 (8.3)	2 (14.3)	3 (27.3)	8 (40.0)	8 (36.4)	22 (27.8)
Median PFS, weeks (95% CI)	5.6 (5.3–6.1)	5.6 (4.4–10.7)	11.9 (8.1–24.4)	6.9 (6.0–11.1)	7.8 (4.0–13.0)	6.9 (6.0–8.1)
Median OS, weeks (95% CI)	-	-	-	6.6 (NE–NE)	15.4 (7.6–32.3)	15.4 (7.1–32.3)

[a] Tumor response was measured using RECIST 1.1 (17), with tumor assessment every 2 cycles during the treatment period for each schedule in all patients with solid tumors. For patients with mCRPC without measurable disease according to RECIST 1.1, tumor response was measured using PCWG3 recommendation (18) every 4 cycles, based on assessment by bone scan coupled with percentage change in prostate-specific

antigen at the same time points as the bone scan. For Schedule B in the DLBCL cohort, tumor response was assessed using RECIL 2017 (19) based on FDG-PET/CT scans every 2 cycles during first 4 cycles, then every 4 cycles. The assessment of overall response rate in DLBCL only included patients who achieved a partial or a complete response.

[b] Objective response is defined as best overall response of complete response or partial response with tumor assessment during treatment period for each schedule.

[c] Disease control is defined as best overall response of complete response, partial response, or stable disease.

CI, confidence interval; CRC, colorectal cancer; CT, computerized tomography; DLBCL, diffuse large B-cell lymphoma; FDG-PET, fluorodeoxyglucose-positron emission tomography; mCRPC, metastatic castration-resistant prostate cancer; NE, not evaluable; NUT, nuclear protein in testis; OS, overall survival; PCWG3, Prostate Cancer Working Group 3; PFS, progression-free survival; RECIL, Response Evaluation Criteria for Lymphoma; RECIST, Response Evaluation Criteria in Solid Tumors; SCLC, small-cell lung cancer.

Supplementary Table 4. Non-compartmental PK parameters [gMean (gCV%)] after single and multiple oral administration of BI 894999, Cycle 1, by study Phase, dose schedule, dose, and indication

**Parameter [Unit]**	**$AUC_{0–24}$ [nmol·h/L]**	**$AUC_{0–24,ss}$ [nmol·h/L]**	**C_{max} [nmol/L]**	**$C_{max,ss}$ [nmol/L]**	**t_{max} [h]**	**$t_{max,ss}$ [h]**
**Dose group**	**gMean (gCV, %)**	**gMean (gCV, %)**	**gMean (gCV, %)**	**gMean (gCV, %)**	**Median (Min – Max)**	**Median (Min – Max)**
**Phase Ia extension – DLBCL**						
**Schedule B**						
1.5 mg	n=7<br>31.7 (33.4)	n=6<br>70.3 (38.5)	n=7<br>2.98 (34.9)	n=6<br>5.48 (32.7)	n=7<br>2.02<br>(1.00–4.00)	n=6<br>2.56 (1.00–3.03)
2.0 mg	n=4<br>48.0 (26.9)	n=3<br>120 (19.6)	n=4<br>4.49 (36.0)	n=3<br>7.67 (8.44)	n=4<br>2.01<br>(2.00–3.00)	n=3<br>2.12 (1.00–6.00)
2.5 mg	n=2<br>53.3 (6.91)	–	n=2<br>4.75 (0.60)	–	n=2<br>2.04<br>(2.00–2.08)	–
**Schedule C[a]**						
4.0/2.0 mg	n=2<br>138 (7.52)	–	n=2<br>13.9 (12.8)	–	n=2<br>2.00<br>(1.00–3.00)	–
**Phase Ib expansion – solid tumors**						
**Schedule B**						
2.0 mg	n=9<br>39.2 (38.8)	n=10<br>73.6 (58.2)	n=9<br>4.10 (37.8)	n=10<br>6.51 (70.0)	n=9<br>2.00<br>(1.00–3.00)	n=10<br>2.00 (0.93–2.08)
**Schedule B – including NUT carcinoma**						
2.5 mg	n=42<br>67.6 (50.9)	n=33<br>125 (50.4)	n=44<br>6.01 (59.6)	n=33<br>10.4 (59.4)	n=44<br>2.01<br>(1.00–8.00)	n=33<br>2.00 (0.83–8.00)
**Schedule B – NUT carcinoma only**						
2.5 mg	n=17<br>56.9 (58.6)	n=14<br>94.9 (43.9)	n=17<br>4.88 (50.1)	n=14<br>7.68 (45.4)		
**Schedule C – NUT carcinoma only[a]**						
6.0/3.0 mg	n=2<br>214 (6.01)	n=11<br>149 (56.7)	n=15<br>18.8 (55.8)	n=11<br>11.2 (60.1)	n=15<br>2.00<br>(1.97–4.00)	n=11<br>2.05 (2.00–6.00)

[a] No descriptive statistics were prepared for the 5.0/2.5mg (Schedule C – Phase Ia DLBCL) and 7.0/3.5 mg (Schedule C – Phase Ib NUT carcinoma) groups because only one patient was available in each group.

$AUC_{0–24}$, C_{max}, and t_{max} estimated on Day 1 for all schedules (after first dose for Schedule B; after loading dose for Schedule C); $AUC_{0–24,ss}$, $C_{max,ss}$, and $t_{max,ss}$ estimated on Day 14 for Schedule B (after 2 weeks of daily administration) and on Day 21 for Schedule C (after 6 days on maintenance dose).

$AUC_{0–24}$, area under the plasma concentration–time curve over the 24-hour dosing interval; C_{max}, maximal exposure concentration; DLBCL, diffuse large B-cell lymphoma; gCV, geometric coefficient of variation; gMean, geometric mean; NUT, nuclear protein in testis; ss, steady state; t_{max}, time to reach maximal plasma.

Supplementary Table 5. Molecular profiling data

Patient ID	Disease	Known somatic short variants	Likely somatic short variants
852	Colon adenocarcinoma	TP53_c.1045G>T_p.E349*, APC_c.3980C>G_p.S1327	None
471	Colon adenocarcinoma	TP53_c.529_546delCCCCACCATGAGCGCTGC_p.P177_C182del, SMAD4_c.1612G>T_p.E538	RNF43_c.1A>G_p.M1V
527	Colon adenocarcinoma	PIK3CB_c.1654G>A_p.E552K, APC_c.4348C>T_p.R1450, KRAS_c.183A>T_p.Q61H, SMAD4_c.1066C>T_p.P356S	PTEN_c.210-2A>G_p.splice site 210-2A>G, APC_c.2097G>A_p.W699
716[a]	Colon adenocarcinoma	KRAS_c.38G>A_p.G13D, TP53_c.524G>A_p.R175H	None
779[b]	Colon adenocarcinoma	TP53_c.584T>C_p.I195T, NRAS_c.35G>A_p.G12D	LRP1B_c.746G>A_p.W249
732	Colon adenocarcinoma	KRAS_c.34G>T_p.G12C, TP53_c.743G>A_p.R248Q, APC_c.2977A>T_p.K993, FAM123B_c.1057C>T_p.R353, SPTA1_c.4771C>T_p.H1591Y	None
654	Colon adenocarcinoma	APC_c.4724_4726TAG>AA_p.L1575fs*75, APC_c.2626C>T_p.R876, TP53_c.841G>C_p.D281H, KRAS_c.436G>A_p.A146T	None

[a] Patient 716 tumor sample had likely functional rearrangements (e.g., gene fusions) (gene1_gene2_genomic event description_supporting-reads): ARID1B_ARID1B_deletion, RAD51C_CDH9_truncation

[b] Patient 779 tumor sample also had the following non-focal lower-level (cn<=8) amplifications of genes known to be recurrently amplified in cancer: AURKA_amplification, GNAS_amplification, BCL2L1_amplification, ZNF217_amplification, TOP1_amplification, SRC_amplification, ARFRP1_amplification

The tumor sample also had a homozygous deletion of PTEN.
